# Transtheoretical Model of Change during Travel Behavior Interventions: An Integrative Review

**DOI:** 10.3390/ijerph14060581

**Published:** 2017-05-30

**Authors:** Margareta Friman, Jana Huck, Lars E. Olsson

**Affiliations:** SAMOT/CTF Service Research Center, Department of Social and Psychological Studies, Karlstad University, Karlstad 651 88, Sweden ; jana.huck@kau.se (J.H.); lars.e.olsson@kau.se (L.E.O.)

**Keywords:** health, integrative review, transtheoretical model of change, TTM, travel behavior, travel interventions

## Abstract

This study aims to identify the relevant empirical work, to synthesize its findings, and to thus attain a general understanding of the application of the Transtheoretical Model (TTM) in transport behavior research. An integrative literature review was used to determine whether or not the implemented interventions impact the stages and processes of travel behavior change. Data was collected from different databases. English language articles published between 2002 and 2017 were included. After sequentially narrowing the search and removing duplicates, 53 relevant papers remained, 13 of which fulfilled the stated criteria of constituting a transport intervention study using the TTM as a reference frame. The final 13 studies were classified and categorized according to stages and processes in the TTM. Findings showed that none of the interventions met the method requirements for a proper evaluation of design and outcome measurement. Reporting did not follow a standardized structure desirable when enabling comparative analyses. Allowing for these shortcomings, it is inferred that positive travel behavior changes have been obtained during some interventions. Importantly, although it was stated that the empirical studies were based on the TTM, the included interventions were implemented irrespective of the individual’s stage of change. For future research, it will be necessary to conduct evaluations of higher quality.

## 1. Introduction

This review focuses on travel behavior change interventions and the Transtheoretical Model of Change (TTM) [1,2], a process theory of motivation as the foundation of behavioral change. The review will identify and evaluate the relevant empirical work that has applied the TTM, or parts of the theory, to travel behavior intervention programs. The overall aim is to understand whether, and to what extent, the implemented interventions impact the stages and processes of travel behavior change. The general concepts of the TTM will be explained first to provide a basic understanding of the theory, where it has its roots, and in what contexts it has previously been applied. Then follows an integrative review [3] of the TTM and travel behavior interventions, as well as the categorizations of these findings. Finally, applications of the TTM to travel behavior change interventions will be discussed, followed by challenges and avenues for future research.

### The Transtheoretical Model of Change

Prochaska and DiClemente’s [1] Transtheoretical Model of Change is well established in health research [4,5], as well as being used in travel behavior research [6,7]. Compared to attitude/intention-based behavioral models like the Theory of Planned Behavior [8], the TTM describes behavior change as a sequence of the stages through which individuals progress toward a desired kind of behavior. The TTM consists of two main constructs: the stages of change and the processes of change. The popularity of the TTM is based on its possibility of tailoring interventions to individuals undergoing different stages of change.

The TTM consists of five major stages, as previously described by transport researchers [9,10,11] and illustrated in Table 1. During the first stage—precontemplation—the individual has no intention of changing his/her behavior and is unaware of the negative consequences of his/her current behavior. Alternatively, he/she believes that these consequences are insignificant. During the next stage—contemplation—the individual is starting to think about changing his/her behavior within the next six months. However, while contemplating, the cost of change is overestimated and the person remains undecided regarding the benefits. During the ensuing preparation stage, the individual is planning to make a change within a month, and has begun taking small steps towards changing. When people reach the action stage, they have recently changed their behavior and are actively trying to modify their (problem) behavior, and to acquire new behaviors. Finally, individuals transition to the maintenance stage once they have been able to maintain a change for more than six months, and are actively trying to prevent a relapse. Relapsing means regressing by one or more stages, which may occur at any stage [6].

Behavior is a consequence of a set of processes operating on a number of determinants that can be described as cognitive, affective, and/or behavioral, and assumed to facilitate (or trigger) a transition from one specific stage to the next (see Table 1) [12,13]. Interventions, on the other hand, are distinctive measures targeting a specific process. As an example, consciousness raising is a process while interventions for consciousness raising can consist of posters and/or other printed materials. The processes described in Table 1 represent an extended version of the original ten processes identified by Prochaska and DiClemente [1,13]. The processes listed in Table 1, e.g., goal setting, trying new behaviors, and consciousness raising, have all been implemented and tested in health-related research areas, e.g., smoking cessation and condom use [2,13,14,15,16,17]. Depending on the individual stage of change, matching processes are proposed for supporting the individual’s transition to the next stage. For instance, the process of consciousness raising can trigger people into moving from the precontemplation stage to the contemplation stage and, during the process of dealing with different barriers, individuals can move from the preparation stage to action. Below, there is a description, based on the identification of Bartholomew et al. [12], of linkages between the stages and processes of change (see Table 1), including examples of interventions targeting a specific process and triggering a transition.

The process of change, i.e., moving from the precontemplation stage to the contemplation stage, involves *consciousness raising*; that is, finding and learning about new facts, ideas, and ways of encouraging behavior change. The aim is to make individuals aware of the need to alter their current behavior. *Dramatic relief* can be used to produce enhanced (negative) emotional experiences, followed by reduced affect for the undesired behavior. Interventions used to stimulate dramatic relief include role-plays, personal testimonies, and media campaigns. *Environmental reevaluation* includes an evaluation of the impact of an undesired behavior on the social environment. Interventions initiating such an evaluation include empathy training, family interventions, and documentaries on the social environment. Both qualitative and quantitative risks are associated with behavior change. *Risk assessment* involves increased knowledge of: (1) risk comparison (e.g., comparing current negative behavior with future positive behavior) and (2) risk assessment (transactional/short-term probabilities versus cumulative/long-term probabilities of success). Positively-framed interventions encourage a focus on successful behavioral change rather than on failure, likelihood information, personalized messages, and/or facts about the effects of a specific behavior on other people’s aims to alter their risk perception. The process of altering an individual’s *perception of the benefits* of changed behavior includes interventions focusing on the advantages of the desired behavior and the disadvantages of the (current) risk behavior.

The transition from the contemplation stage to the preparation stage, or even to action, is dominated by cognitive and affective experiential process types. The process of *self-reevaluation* includes interventions emphasizing that behavioral change is an important part of the individual’s identity, which is basically an assessment of one’s own self-image (e.g., what one wants to be). Interventions like value clarification, healthy role models, and imagery can trigger an individual’s self-reevaluation. Another process during this transition is *social support and self-efficacy* (interventions allowing skill training), helping individuals cope with the emotional disadvantages perceived to be the result of change. *Decision-making* focuses on supporting the individual’s process of deciding to change. The *tailoring of time horizons* includes interventions adjusting the period of time during which the change is to be carried out by the individual. *Focusing on important factors* emphasizes that aspects of the greatest importance, e.g., important beliefs or consequences, are to be considered. The processes of *trying out new behaviors* (interventions that can facilitate experiences), *persuasion of positive outcomes* (interventions altering expectations), and *modeling* (interventions highlighting role-models who have previously overcome difficult barriers) are aimed at increasing the likelihood of actually making the change.

The transition from preparation to action and from action to maintenance requires behavioral processes in order to facilitate change. For example, *self-liberation* involves making a firm commitment to change (e.g., a New Year’s resolution or signing a pledge). *Skill improvement* refers to changing the existing environment in order to reinforce important, obvious, and socially-supported clues. In order not to relapse, it is important to be able to *cope with barriers*. During this process, barriers are identified and solutions as to how these can be circumvented are developed. *Goal setting* is an effective process for imposing short- and long-term behavioral change since the individual sets specific and incremental goals. Also highlighting role models creates social reinforcement of the changed behavior (*modeling*) to the transition to the action stage.

When reaching the action stage, *helping relationships* can be used to maintain a behavior. *Counter conditioning* is an intervention that focuses on changing responses to a stimulus. This could mean helping people to react more negatively to something they used to react favorably to (e.g., cigarettes). *Contingency management* involves rewarding (or punishing) a behavioral change. *Stimulus control* is a term used to describe situations in which a behavior is triggered by the presence or absence of a stimulus (e.g., if one always eats while watching TV, the eating behavior will be controlled by the stimulus of watching TV). *Skill enhancement* is aimed at reminding people that setbacks occur and at providing tools for overcoming such situations. *Self-rewards* are interventions whereby a feeling of success is promoted in order to remind the individual of the positive consequences. When the individual has reached the maintenance phase, the process of *coping skills* can be used to prevent a future relapse by identifying high-risk situations, developing solutions, practicing these solutions, and coping with setbacks in such a way that the new behavior is maintained.

Over the years, the TTM has become one of the dominant stage models. However, the TTM has also been criticized [6,18,19,20] for its lack of empirical applicability, for not being clear and consistent regarding which factors influence the transition to the next stage, and for its lack of clarity when it comes to explaining how and why people change. Further, it is argued that the TTM has not been validated when it comes to stage-matched interventions versus mismatched interventions [20]. One of the main arguments for TTM is its ability to design tailored and individualized interventions. Sutton [20] emphasizes that this supposedly differentiating factor not only applies to stage models but also to continuum models like the theory of reasoned action [21]. Even though the TTM has been criticized, it is, nonetheless, popular when it comes to explaining behavioral change in various contexts, e.g., health-related behavior [22,23], environmental behavior [4], organizational change [24], and consumer behavior [25,26]; however, relatively little is known of its applicability in relation to travel behavior change. In the present integrative review, our focus is on the use of the Transtheoretical Model of Change in transport, summarizing the findings and identifying the processes found to support and/or inhibit travel behavioral change.

## 2. Materials and Methods

### 2.1. Integrative Review

Due to the varied mix of research designs and application of the TTM in the transport domain, an integrative review approach has been chosen [3]. Consistent with a systematic framework, the stages of the review were: (1) problem identification, as outlined in the introduction; (2) literature search; (3) data evaluation; (4) data analysis (including ordering, coding, categorization and summarizing), and (5) data interpretation and the presentation of findings [3].

### 2.2. Procedure and Inclusion Criteria

Studies were identified by searching in the international research literature on the Transtheoretical Model of Change as used in travel behavior research from 2000 to 2017. We conducted an electronic database search that included Google Scholar, Web of Science, and PsychNET. The databases ScienceDirect and EBSCOhost were consulted when we were redirected there from the previous three databases. Searching in all database fields, we used a search string containing keywords and travel-related keywords (summarized in Table 2). This resulted in 18,300 articles in Google Scholar, 5017 in Web of Science, and 1142 in PsychNET. The result was filtered using the category “transportation and behavioral science” for Web of Science (1392 articles) and the category “Human” for PsychNET (864 articles). These broad filters reduced the number of hits, while also minimizing the risk of missing important papers. Google Scholar could not be filtered and was only sorted by relevance. As the keywords are commonly used terms, the search results contained papers which merely mentioned the TTM or other stage models, or which referred to other contexts than travel.

**Figure 1:**
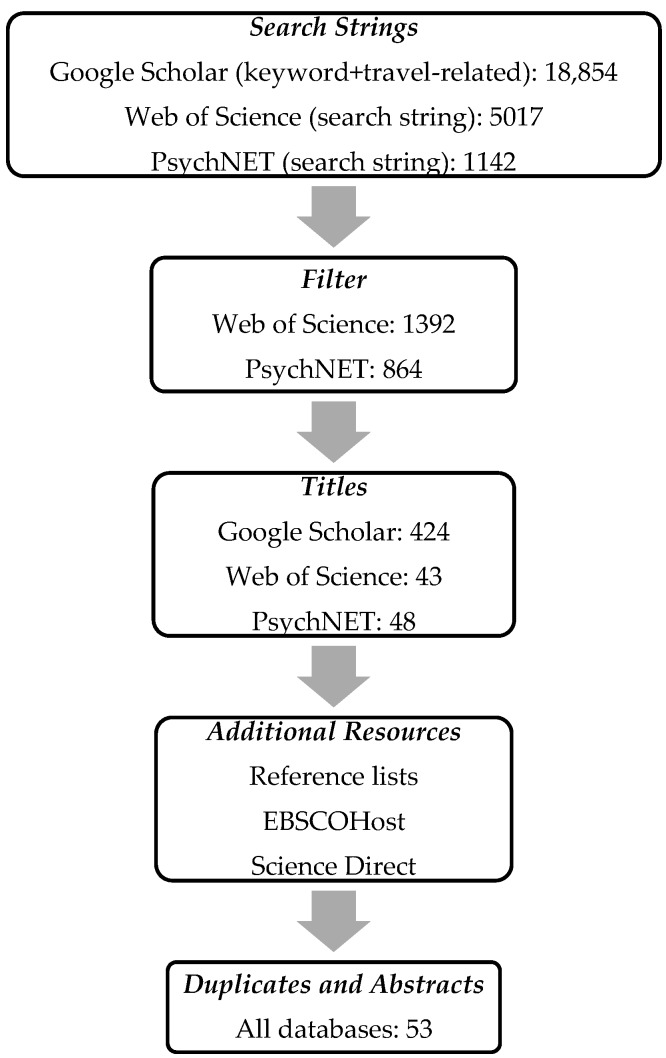
Literature search process.

Papers with relevant titles were included in the first selection (see the literature search process illustrated in Figure 1). Reference lists of the identified papers were consulted to identify additional research findings that had been published.

After removing duplicates and screening for general relevance, 53 articles progressed to a second selection process. During the second selection process, we applied five inclusion criteria. The study had to use the TTM as (1) a theoretical frame, (2) a supporting theory, or (3) an analytical tool. Subsequently, studies using all or just a few of the constructs of the TTM were considered appropriate. A fourth criterion (4) stated that all modes of travel and motives for travel behavioral change were deemed relevant. A fifth criterion (5) dictated that the included studies should contain detailed information about the research methods. However, no restrictions were made regarding the type or quality of the study design. Literature reviews and theoretical papers were later separated from empirical studies. After a thorough and incremental selection process, 38 studies were selected and classified with regard to the type of study: literature review (n = 5), theoretical paper (n = 3), or empirical study (n = 30). Finally, 13 intervention-based studies were selected on the basis of their reporting of outcome measures in relation to the TTM (see Table 3). Studies with an empirical and non-intervention-based design were summarized, also serving as supportive material.

## 3. Findings

During the first stage of analysis, the included papers are classified and categorized. We then present how the processes and stages have been applied and what interventions have been implemented in order to facilitate behavioral change. The third and final section presents and analyzes the different outcome measures.

### 3.1. Classification and Categorization

Table 3 summarizes the included studies by transport mode, motive, target group, method(s), number of participants, time frame, country, travel habits, target behavior, use of the TTM, and findings. The total number of participants involved in the interventions varied between 22 and 1952 (M = 559 participants; SD = 553). One study did not report the number of participants. The interventions lasted between 1 day and 24 months (M = 7.06 months; SD = 7.53). The compilation of studies showed that interventions had been conducted in different countries, although studies from the English-speaking countries dominated (USA, Australia, UK, and Canada).

One third (n = 4) of the identified studies had been carried out in a university setting that included both staff and students. Three studies had focused on specific communities or neighborhoods, while three other studies had been conducted at workplaces, for instance in the metallurgy industry or in the public sector. Other settings were schools and special events.

Each intervention study specified an overarching motive. Two dominant motives for implementing the interventions were increased health and physical activity (n = 6) and an improved environment (sustainability) (n = 2). Some interventions included different combinations of health, physical activity, and environmental motives (n = 5). In addition, the majority of the studies had been published in health-related journals (n = 8), a few in transport journals (n = 3) or in other journals (n = 2).

The majority (n = 10) of the included studies had used a quantitative method, with three using a mix of qualitative and quantitative methods. Eleven of the intervention studies were quasi-experimental while seven included a control group. Twelve studies included self-reported measures while one contained observational data.

The target groups were employees (n = 4), students (n = 3), car drivers (n = 2), the citizens of a predefined neighborhood (n = 1), school children (n = 1), and obese women (n = 1).

All the studies but one reported a travel behavior base-line in order to identify and select a target group for the relevant intervention. Car users (n = 5) or mixed mode users (n = 3; car, public transport, biking, walking) were targeted. Few studies targeted users who were not using a pre-specified mode at the time of the intervention (e.g., non-walkers or non-bicyclists).

The target behavior describes the goal of the intervention, e.g., increased biking (n = 8), increased general activity levels (n = 5), increased walking (n = 3), increased public transport use (n = 1), improved attitudes toward active transport (public transport, biking, walking) (n = 1), and reduced car use (n = 1).

### 3.2. Processes and Stages of Change

Table 4 summarizes the processes that have been supported during transport interventions aimed at changing travel behavior. The interventions identified in the selected studies have been sorted in terms of which process the intervention intends to support and in terms of which stage the intervention are implemented. This is reported as frequencies in Table 4. Since an intervention usually supports several processes, the total frequency in Table 4 adds up to a higher frequency than the included 13 studies. To illustrate the coding process, an information campaign could be coded in support of consciousness raising and positive framing. The frequency also indicates during which stage the intervention was implemented. For instance, interventions supporting the consciousness-raising process have been used twice during the precontemplation stage (see Table 4). Thus, the frequencies represent the number of times an intervention has supported a specific process, and during what stage.

Table 4 shows that 21 out of a possible 28 processes have been supported by different interventions in the transport domain. Seven processes were not supported by any of the implemented interventions (e.g., dealing with barriers and skill enhancement).

Looking more closely at each stage, we find that interventions supporting the consciousness-raising process were implemented during all stages, although the theory recommends that such interventions should be implemented during the precontemplation stage. However, few studies have implemented any interventions supporting processes during the precontemplation stage. Participants during the precontemplation stage, which includes individuals not intending to take action in the near future, may not have been the main target group during transport interventions.

During the contemplation stage, interventions supporting 5 out of a possible 8 processes were implemented (e.g., *self-efficacy and social support* and *persuasion of positive outcomes*). However, interventions during this stage were intertwined and thus also supported processes targeting other stages. Contemplators, individuals who intend to take action within the next six months, were supported in their process of trying out new behavior; however, at the same time, they were also supported in their skill improvement even though such support should be provided during the preparation stage, according to the TTM. It is concluded that several interventions during the selected studies were implemented in order to support processes during a non-matching stage. Furthermore, a combination of interventions supporting a number of processes, irrespective of stage, were commonly implemented during transport behavior change interventions. The same pattern was observed during the preparation stage, where individuals are ready, and expected, to take action within the next month.

Much fewer interventions were implemented in support of processes during the action and maintenance stages (e.g., *helping relationships* or *counterconditioning*). Surprisingly, interventions supporting the consciousness-raising process were implemented in the case of participants who had already made a change. In the selected studies, no interventions were implemented in support of coping skills during the maintenance stage.

Interventions supporting a specific process without defining a specific stage were classified as an undefined stage in Table 4. For example, interventions supporting an environmental reevaluation process were implemented during the so-called pre-action stage in several studies. This pre-action stage is described as a combination of the precontemplation, contemplation, and preparation stages and is thus coded as an undefined stage since it does not match any of the original stages in the TTM.

To summarize, Table 4 shows that transport interventions for behavioral change greatly support processes such as consciousness raising, self-efficacy, social support, and skill improvement. Furthermore, the identified inventions had most frequently been implemented during the contemplation stage, followed by the preparation stage. However, several interventions could not be related to any specific stage of change, and were thus classified as an undefined stage. We were surprised that so few interventions had supported processes relating to the participants’ stages of change. This was surprising, since the TTM was part of the theoretical framework, but had not been used in the design of the intervention. Thus, transport interventions do not follow the general guidelines stated in the theoretical framework, where specific interventions are recommended in support of certain processes in order to facilitate a change of stage. When interventions are bundled into one offer, e.g., a self-help package in combination with group activities, this results in a mix of interventions supporting different processes in a state of mismatch with the corresponding stage of change.

### 3.3. Outcome Measures

#### 3.3.1. Travel Behavioral Change

A consistent finding is the lack of comparable outcome measures related to the stage of change. Three studies report data relevant to the stage of change, but no conclusions could be drawn due to reporting being too abstract [27], no post-intervention reporting [34], or no differentiation between transport modes [10]. Rose and Marfurt [34] reported an overall stage progression (85% of the participants progressed to a higher stage), but did not relate this progression to specific stages. Yet another study, by Mundorf et al. [10], reported that 21% of the participants undergoing the precontemplation stage moved to either the contemplation stage (16%) or the preparation stage (4%) after a computer-based intervention. A number of studies [28,30,33] presented changes without relating these to a specific stage in the TTM, instead using a merged category which included several stages (pre-contemplation, contemplation and preparation) and defining it as a pre-action stage. Although it does not relate the travel behavioral change to a specific stage, Table 5 summarizes the general outcome measures (willingness to use a target mode, trips per mode, main mode use, travel duration, and travel distance) reported in the included papers. Whenever possible, calculations were based on the reported raw data, e.g., number of trips, number of meters, and steps. As the number of steps was only reported in one study, this data was converted into travel distance using an average step length of 0.69 m [37].

As can be seen in Table 5, four studies reported an increased willingness to use a specific mode post-intervention [28,30,33,34], two with respect to bike use, one with respect to walking, and one with respect to a combination of these modes (active transport). Four studies reported a change in the number of trips per transport mode [28,31,33,35] of which three reported a decrease in car trips, two reported an increase in public transport trips and/or trips by bike, and there was one each for increased walking or carpooling. Changes in the main transport mode were reported in five of the studies [9,31,34,35,36], showing an increase in bicycle use, walking, and the use of active transport modes in combination. Car use and public transport use were reported as decreasing due to the intervention. Two studies reported changes in travel time per transport mode [7,33]. Both studies showed that the time travelled by bike and on foot increased. The percentage change in the distance traveled per transport mode was calculated in two of the studies [29,30], showing that bicycling and walking distances had increased while the distance traveled by car had decreased.

#### 3.3.2. Other Outcome Measures

Eight out of 13 intervention studies reported changes in attitudes toward travel. Gatersleben and Appleton [28] reported changes in attitudes toward travel modes during different stages. They found that, as people progressed into higher stages, they reported more positive attitudes toward cycling. In the other seven studies, attitudes were not reported with respect to stage changes but with respect to specific transport modes [30,33,35]. Three studies reported general changes in health and weight or BMI [7,29,32]. However, significant health improvements due to interventions were only reported with regard to a general change in health [7]. No changes were noted in weight or BMI.

The outcome measures reported in the social-media-based intervention [36] differed from all the other studies. Outcome measures were reported in terms of average click rates, likes, and shares of the intervention material posted on Facebook and Twitter. While the findings showed that certain posts/activities generate higher levels of engagement on social media, no data was reported regarding the stages of change or the actual travel behavior.

#### 3.3.3. Summary Outcome Measures

In summary, the included studies focused on different target groups, behaviors, and settings. Car users and the staff and students of universities were common target groups. The effect of the interventions could not be ruled out since these generally supported a combination of processes instead of a single process. The included studies were reported before and after the outcome measures in relation to a specific transport mode, or in relation to the share per transport mode. The number of trips, users per transport mode the distance travelled, and the travel duration were all reported. The units in which the outcomes were measured varied, thus making comparison difficult, especially when raw data was not provided. Surprisingly, only three studies focused on the progression to higher stages of change [9,27,31]. Four studies reported an improved willingness to use the target transport mode, reflecting a progression during the pre-action stages [28,30,33,34]. A few studies reported a more positive attitude toward the target behavior [30,34], or a slight improvement in health due to the intervention [7,29,32].

## 4. Discussion

The purpose of the present study was to conduct an integrative review aimed at attaining a general understanding of the application of the Transtheoretical Model (TTM) of Behavioral Change in the transportation domain. The study thus included quantitative and qualitative empirical papers describing travel behavior interventions either based on or related to the TTM. The findings show that several interventions have been conducted in different countries aiming to transform a specific travel behavior by applying stages of change. It has been difficult, however, to find empirical studies that have included any outcome measures in relation to the processes and stages of change defined in the TTM. In addition, the mapping of the processes and the stage of change varied greatly, making evaluations and comparisons difficult. Of the 53 papers found, only 13 contained sufficient information to enable analysis.

The majority of the implemented interventions support several processes for facilitating the stage of change. Examples include interventions supporting consciousness raising [9,28] which were implemented during all stages, as well as interventions supporting skill improvements [7,29], which were implemented during the precontemplation and contemplation stages. The common mismatch between processes and stages of change emphasizes the need for further research. Seven processes (i.e., decision-making perspective, modeling to overcome barriers, modeling perception/social reinforcement, stimulus control, skill enhancement, dealing with barriers and coping skills) were identified as not being supported by any interventions; a question arising here is whether they are unsuited to travel behavioral interventions or whether they have not been used due to a lack of knowledge of their potential efficiency in this area.

The selected studies confirm that interventions supporting various processes defined in the TTM successfully trigger a change in travel behavior. The positive changes concern a reduction in car travel (number of trips) and an increase in trips using active modes (e.g., public transport, bicycle trips, and walking). Also, carpooling increases in the number of trips made. Changes in willingness to use a specific mode, actual main mode use, and trip distance in meters per day all followed the same pattern. The most common motive for implementing interventions is related to health and travel, and examples include “Walk in to Work out” [7] and “Bicycle-sharing program” [32], where several people aimed to increase their general physical activities in daily life. Health was also observed as an outcome measure (e.g., expected health improvements, weight, or BMI), although no significant effects were observed.

The included studies provide very limited knowledge of the way in which transport interventions support single processes related to specific stages of the TTM. Investigating this relationship, and how it can contribute to travel behavioral change, would not only strengthen the applicability of the TTM to travel research, it would also provide important insights into how individuals can be encouraged to change their travel behavior. Some studies [27,31,32,33,36] combined several processes (precontemplation, contemplation, and preparation) during a so called pre-action stage. As a consequence, it becomes even more difficult to draw conclusions about how interventions supporting single processes have affected the progression from stage to stage. Clearly, identifying interventions that lead to changes in travel behavior will necessitate more comprehensive evaluation studies that will examine both the process and the stage of change in order to determine which processes are the most important ones to support, and during which stage in order to achieve positive and effective outcomes. Such research will increase our ability to draw conclusions about how specific processes influence outcomes.

The most common setting for TTM studies is universities and other public institutions, thus limiting comparability, since the majority of the participants are highly educated, while low education/income groups go underrepresented. The selected settings are generally equipped with high quality infrastructure and facilities that support alternative travel. For instance, there are lighted cycle lanes, shelters, and safe lanes that support both pedestrians and cyclists in campus areas. A question that arises is to what degree the reported findings are easily transferable to settings with poorer infrastructure (e.g., where cycling lanes are mixed with other types of traffic).

The variability of the quality of the studies, including the study designs, the range of sample sizes, the measures used, and the duration of the interventions (i.e., a day, a couple of weeks, or even a couple of months), limits our ability to make comparisons. Furthermore, the lack of scientific knowledge makes it difficult to make specific recommendations about effective interventions promoting altered travel behavior based on the Transtheoretical Model of Behavioral Change. In future studies, one could consider the potential that technology-based interventions can have to support effective travel interventions [38,39]. We need to take the criticism leveled at the model seriously, and to investigate it further. Care must be taken for the intervention to be adequately implemented, documented, and properly evaluated. The health psychology domain provides intervention developers with guidelines that can be of use to the transport domain as well. An iterative protocol may help in gathering information so that a cumulative science of travel behavioral change, based on the Transtheoretical Model, can be developed [40,41].

## 5. Conclusions

This study demonstrates how travel behavior changes can be obtained with different types of interventions. Interventions relating to processes and stages of change are presented. However, in order to conclude about their effectiveness in transport behavior research more effort is needed to include proper designs and outcome measures in relation to the processes and stages of change defined in the Transtheoretical Model (TTM).

## Figures and Tables

**Table 1:** Overview of the stages and processes of change (Adapted from “Planning Health Promotion Programs: An intervention mapping approach.” by Bartholomew et al. [12]).

Processes	Stages				
	Precontemplation	Contemplation	Preparation	Action	Maintenance
Consciousness Raising	x				
Dramatic Relief	x				
Environmental Reevaluation	x				
Risk Assessment	x				
Positive Framing	x				
Reevaluation of Outcomes	x				
Perception of Benefits	x				
Self-Reevaluation		x			
Self-Efficacy and Social Support		x			
Decision-Making Perspective		x			
Tailoring Time Horizons		x			
Focus on Important Factors		x			
Trying New Behavior		x			
Persuasion of Positive Outcomes		x			
Modeling (Overcoming Barriers)		x			
Self-Liberation			x		
Skill Improvement			x		
Coping with Barriers			x		
Goal Setting			x		
Modeling (Social Reinforcement)			x		
Helping Relationships				x	
Counterconditioning				x	
Contingency Management				x	
Stimulus Control				x	
Skill Enhancement				x	
Dealing with Barriers				x	
Self-Rewards for Success				x	
Coping Skills					x

**Table 2:** Keywords and travel-related keywords used in the review.

Keyword	Context (Travel-Related Keywords)
TTM	Travel
Stage of change	Transport
Stages of change	Transit
Behavioral change	Cycling
Transtheoretical Model	Walk
Stage Model of Change	Car
	Physical activity
	Active commuting

**Table 3:** Overview of the included intervention studies.

Author(s)	Transport Mode	Motive	Target Group	Methods	n	Time	Country	Current Mode use	Target Behavior	Use of the TTM	Findings
Cooper (2007) [27]	Car Public Transport	Environment Health	Local community	Quasi-experimental Quantitative Intervention	1031	10 weeks	USA	Habitual car user	Reduced car use	Classification/stages	Stage of change transition
Diniz et al. (2015) [9]	Bike	Health Physical activity	Workplace Production industry	Quasi-experimental Quantitative Controlled Intervention	932	6 months	Brazil	Non-bikers	Increased biking	Tailored intervention	Change of commuting behavior
Gatersleben & Appleton (2007) [28]	Bike	Sustainability	University	Quasi-experimental Quantitative Intervention (Study 1) Survey (Study 2)	89 (Study 1) 22 (Study 2)	2 weeks	UK	Car and public transport users, walkers	Increased biking	Theoretical framework Categorization/strategies	Stage of change transition Motivators and barriers
Hemmingsson et al. (2009) [29]	Bike Walk	Health Physical activity	Workplace Health care	Quasi-experimental, Quantitative Controlled Intervention	120	18 months	Sweden	Car and public transport users	Increased everyday activity levels	Tailored intervention	Stage of change transition
McKee et al. (2006) [30]	Car Walk	Health Physical activity	School (9–10 years)	Quasi-experimental Mixed Controlled Intervention	60	10 weeks	UK	Habitual car users	Increased walking	Tailored intervention	Stage of change transition Increased walking
Meloni et al. (2013) [31]	Car Public Transport	Environment Sustainability	Local community	Descriptive Quantitative Survey and Intervention	146	1 week	Italy	Habitual car users	Increased public transport use	Theoretical framework Classification/stages	Model for voluntary change
Molina-Garcia et al. (2013) [32]	Bike	Health Physical activity	University	Cross-sectional, Quantitative Survey and intervention	173	8 weeks	Spain	Car/motorbike/public transport users. Walkers and cyclists	Increased biking	Analytical tool Classification/stages	Increased bike-rentals
Mundorf et al. (2013) [10]	Public Transport Bike Walk	Environment Physical activity	University	Quasi-experimental Descriptive Quantitative Survey and Intervention	588 (Study 1) 1196 (Study 2) 720 (Study 3) 393 (Study 4)	1 day	USA	Habitual car users	Increased use of alternative transport	Study design Analytical tool Stages of change Decisional balance Self-efficacy Processes of change	Stage of change transition pre-action
Mutrie et al. (2002) [7]	Bike Walk	Health Physical activity	Workplace Public sector	Quasi-experimental Descriptive Survey Controlled intervention	295	12 months	UK	Habitual car users	Increased active commuting	Study design Analytical tool Stages of change Decisional balance Self-efficacy Processes of change	Stage of change transition
Rissel et al. (2010) [33]	Bike	Environment Health	Local community	Quasi-experimental Quantitative Controlled Intervention	909	24 months	Australia	Biking	Increased biking	Tailored intervention	Stage of change transition Increased biking
Rose & Marfurt (2007) [34]	Bike	Environment Sustainability	Local community	Quasi-experimental Quantitative Survey Intervention	1952	1 day + 5-day follow-up	Australia	Car, public transport, biking, walking	Increased biking	Predictive and analytical tool	Stage of change transition
Wen et al. (2016) [35]	Public Transport Bike Walk	Health Physical activity	Work place Health care	Quasi-experimental Mixed Controlled Intervention	68	12 months	Australia	Car, active transport (public transport, biking, walking)	Increased physical activity levels Increased biking Increased walking	Tailored intervention	Increased use of active transport. Commuting and leisure activities
Wilson et al. (2011) [36]	Bike	Physical activity	University	Descriptive Quantitative Observational intervention-based study	280 followers	12 months	USA	Not measured	Increased physical activity levels	Tailored intervention	Increased social media use

**Table 4:** Frequency of interventions supporting specific processes and stages of change.

Stages of the TTM	Processes (Total 28)	Stages of the TTM					Undefined Stage	Process Not Supported
		Precontemplation	Contemplation	Preparation	Action	Maintenance		
**Precontemplation**	Consciousness raising	2	11	25	3	3	15	
	Dramatic relief		3	7			5	
	Environmental reevaluation		1	6			5	
	Risk Assessment		2	3			1	
	Positive framing		3	3			1	
	Reevaluation of outcomes		2	2				
	Perception of benefits		1	1			1	
**Contemplation**	Self-Reevaluation			3			2	
	Self-Efficacy and Social Support		3	12			7	
	Decision-Making Perspective							x
	Tailoring Time Horizons		1					
	Focus on Important Factors		1				1	
	Trying New Behavior		1	6			4	
	Persuasion of Positive Outcomes		2	7			3	
	Modeling to Overcome Barriers							x
**Preparation**	Self-Liberation		1	5			2	
	Skill Improvement		7	16			8	
	Coping with Barriers		1					
	Goal Setting		1	1			1	
	Modeling Perception/Social Reinforcement							x
**Action**	Helping Relationships			1	1	1	1	
	Counterconditioning		2	2	1	1		
	Contingency Management				2	2		
	Stimulus Control							x
	Skill Enhancement							x
	Dealing with Barriers							x
	Self-Rewards for Success		1					
**Maintenance**	Coping Skills							x
**Summary**		2	44	100	7	7	57	7

**Table 5:** A summary of the general outcome measures post-intervention in the included papers.

	Car	Public Transport	Bicycle	Walk	Car Pool	Active Transport
**% change in willingness to use a specific mode (n = 4)**						
**n ^a^**	0	0	2	1	0	1
**M**			48%	72%		19%
**SD**			20%	na		na
**Min/Max**			29/68	na		na
**% change in number of trips (n = 4)**						
**n ^a^**	3	2	2	1	1	0
**M**	−16.73	29.15	50.45	41	44	
**SD**	7.56	19.15	30.55	na	na	
**Min/Max**	−27.3/−10	10/48.3	19.9/81	na	na	
**% change in main transport mode use (yes/no) (n = 5)**						
**n ^a^**	3	2	4	2	0	1
**M**	−6.97	-14.80	10.93	8.30		7.8
**SD**	0.05	0.1	0.12	0.09		na
**Min/Max**	−13.1/0	−25/−4.6	−0.6/29.4	−0.5/17.1		na
**% change in time travelled per transport mode (minutes/day/person) (n = 2)**						
**n ^a^**	0	0	2	1	0	0
**M**			11.75	52		
**SD**			0.12	na		
**Min/Max**			0/23.5	na		
**% change in distance traveled per transport mode (meters/day/person) (n = 2)**						
**n ^a^**	1	0	1	2	0	0
**M**	−54		38	153.21		
**SD**	na		na	1.37		
**Min/Max**	na		na	16.53/289.9		

^a^ Number of studies reporting the outcome measure.
